# Riboflavin-Ultraviolet A Corneal Cross-linking for Keratoconus

**DOI:** 10.4103/0974-9233.58418

**Published:** 2009

**Authors:** Tamer M. El-Raggal

**Affiliations:** Department of Ophthalmology - Ain Shams University - Cairo, Egypt

**Keywords:** Crosslinking, Keratoconus, Riboflavin

## Abstract

**Purpose:**

To evaluate the safety, efficacy of riboflavin-ultraviolet A irradiation (UVA) corneal cross-linking and present refractive changes induced by the treatment in cases of keratoconus.

**Materials and Methods:**

The study includes 15 eyes of 9 patients with keratoconus with an average keratometric (K) reading less than 54 D and minimal corneal thickness greater than 420 microns. The corneal epithelium was removed manually within the central 8.5 mm diameter area and the cornea was soaked with riboflavin eye drops (0.1% in 20% dextran τ-500) for 30 minutes followed by exposure to UVA radiation (365 nm, 3 mW/cm^2^) for 30 minutes. During the follow-up period, uncorrected visual acuity (UCVA), best spectacle-corrected visual acuity (BSCVA), manifest refraction, slit lamp examination and topographic changes were recorded at the first week, first month, 3 and 6 months.

**Results:**

There was statistically significant improvement of UCVA from a preoperative mean of 0.11 ± 0.07 (range 0.05–0.3) to a postoperative mean of 0.15 ± 0.06 (range 0.1–0.3) (*P* < 0.05). None of the eyes lost lines of preoperative UCVA but 1 eye lost 1 line of preoperative BSCVA. The preoperative mean K of 49.97 ± 2.81 D (range 47.20–51.75) changed to 48.34 ± 2.64 D (range 45.75–50.40). This decrease in K readings was statistically significant (*P* < 0.05). All eyes developed minimal faint stromal haze that cleared in 14 eyes within 1 month. In only 1 eye, this resulted in a very faint corneal scar. Other sight threatening complications were not encountered in this series. Progression of the original disease was not seen in any of the treated eyes within 6 months of follow-up.

**Conclusion:**

Riboflavin-UVA corneal cross-linking is a safe and promising method for keratoconus. Larger studies with longer follow up are recommended.

## INTRODUCTION

Keratoconus is a non-inflammatory progressive disease of the cornea with onset that generally occurs at puberty. Its incidence in the general population is reported to be about 1 in 2000.^1^

Changes in corneal collagen structure,^2^,^3^ and extracellular matrix, apoptosis and necrosis of keratocytes, exclusively involving the central anterior stroma, and Bowman's membrane are documented in the structurally weakened corneal tissue in keratoconus. 4–8

The technique of corneal collagen cross-linking consists of photo polymerization of stromal collagen fibers by the combined action of a photosensitizing substance (riboflavin or vitamin B2) and ultraviolet type A rays (UVA).^9^ Photo polymerization increases the rigidity of corneal collagen.^10^ A similar mechanism of hardening and thickening of collagen fibers has been shown in corneal aging and is related to active glycosylation of age dependent tropocollagen molecules.^11^

The idea to use this conservative approach to treat keratoconus was conceived in the 1990s.^9^ It started with an aim to slow or arrest progression that would delay or avoid keratoplasty. The basis for its use finds clinical and scientific support in the fact that young diabetic patients never have keratoconus due to the natural cross-linking effect of glucose which increases corneal resistance in these patients. The biomechanical properties of cornea depend on the characteristics of collagen fibers,^2^,^3^ interfibril bonds,^5^ and their spatial-structural disposition.^4^ Biomechanical resistance of cornea of keratoconus patients is half that seen in normal corneas. Cross-linking stabilizes stromal collagen, increasing the biomechanical stability of the cornea.^12^

This study aims to assess the safety of riboflavin-UVA induced cross-linking of corneal collagen in reducing the progression of keratoconus and present the visual and refractive changes.

## MATERIALS AND METHODS

Fifteen eyes of 9 patients (6 females and 3 males) with grade I - III keratoconus (according to Amsler- Krumeich classification)^13^ were included in this study. The mean age of the participants was 26.4 ± 3.44 years (range 21–31 years). The inclusion criteria included a completely clear cornea with average keratometric (K) reading Less than 54 D and minimal corneal thickness more than 420 micron with the absence of any other ocular or systemic disease.

The preoperative examination included slit lamp examination, uncorrected visual acuity (UCVA), best spectacle-corrected visual acuity (BSCVA) measurement, corneal topography (TMS- 4, Tomey Inc., Japan), corneal thickness measurement using ultrasonic pachymetry (Nidek Co. Ltd., Japan) and dilated fundus examination.

### Surgical technique

After topical anesthetic eye drops administration, 20% alcohol was applied on the corneal epithelium for 30 seconds. The epithelium was mechanically removed within the central 8.5 mm diameter area using a Beaver blade. Next, riboflavin (0.1% solution, 10 mg riboflavin-5-phosphate in 10 ml dextran*τ* -500 20% solution) (Ricrolin, Sooft Italia, Montegiorgio, Italy) was applied every 3 minutes for 30 minutes until the stroma was completely saturated and aqueous stained yellow.

Ultraviolet, A irradiation, was accomplished using a commercially available UVA system (UVX, Peschke Meditrade, Switzerland). Before treatment, the intended 3 mW/cm^2^ surface irradiance (5.4 J/cm^2^ surface dosage after 30 minutes) was calibrated using a UVA meter (LaserMate-Q, LASER 2000, Wessling, Germany). During treatment, riboflavin solution was applied every 5 minutes to ensure saturation and balanced salt solution (BSS^®^) was applied every 3 minutes to moisten the cornea. Topical anesthetic drops were instilled every 10 minutes throughout the procedure.

A drop of lomefloxacin 0.3% (Okacin, Novartis Ophthalmics) and a bandage contact lens (AcuVue, Johnson and Johnson Vision Care Inc.) were applied at the end of the surgery.

Postoperative treatment included Okacin eye drops 5 times daily for 1 week and Diclofenac 0.1% eye drops (Voltaren^®^, Novartis Ophthalmics) 3 times daily for 1 month. Fluorometholone eye drops (FML^®^, Allergan Inc.) was used twice daily in cases of exaggerated stromal haze.

Follow-up was first done after 3 days for contact lens removal, then after 1 week, 1 month, 3 and 6 months for assessment of UCVA, BSCVA, and slit lamp examination. Refractive and topographic changes were also recorded.

The statistical analysis was carried out using SPSS version 10 for Windows (SPSS Inc., Chicago, IL). Student's *t* test for paired data was used to compare preoperative and postoperative data.

## RESULTS

Table 1

**Table 1:** Preoperative, postoperative means; ranges of the studied parameters, and their statistical changes (*P * values).

	Mean preoperative (Range)	Mean postoperative (Range)	P value
UCVA	0.11 ± 0.07 (0.05–0.3)	0.15 ± 0.06 (0.1–0.3)	0.005
BSCVA	0.51 ± 0.11 (0.4–0.7)	0.53 ± 0.09 (0.4–0.7)	0.189
Sphere (D)	−3.20 ± 1.46 (−1.25–6.25)	−2.73 ± 1.56 (−0.5–−6.0)	<0.001
Cylinder (D)	4.90 ± 0.74 (3.75–6.0)	4.95 ± 0.76 (4.0–6.0)	0.384
K1 (D)	47.77 ± 1.79 (44.8–50.1)	46.27 ± 1.66 (43.6–48.7)	<0.001
K2 (D)	52.17 ± 1.66 (49.2–54.1)	50.42 ± 1.57 (47.9–52.7)	<0.001
Pachymetry (u)	444.0 ± 18.42 (420–480)	446.67 ± 18.39 (420–480)	0.272

shows the preoperative and postoperative means of the studied parameters of the eyes included in the study (UCVA, BSCVA, spherical, cylindrical errors, keratometric readings and pachymetry) and the statistical significance of the changes (*P* values).

### Uncorrected visual acuity

There was a statistically significant improvement from the preoperative values (*P* < 0.05). The preoperative mean UCVA was 0.11 ± 0.07 (range 0.05–0.3) and changed to 0.15 ± 0.06 (range 0.1–0.3). All eyes achieved UCVA of 20/200 or better. In 5 eyes (33.33%) there was 1 line improvement in UCVA. None of the eyes lost lines of the preoperative UCVA.

### Best spectacle-corrected visual acuity

There was no statistically significant change from the preoperative values (*P* > 0.05). The preoperative mean BSCVA was 0.51 ± 0.11 (range 0.4–0.7) and changed to 0.53 ± 0.09 (range 0.4–0.7). All eyes achieved BSCVA of 20/50 or better. In 4 eyes (26.67%), there was 1 line improvement and only 1 eye (6.67%) lost 1 line of preoperative BSCVA.

### Spherical error

There was a statistically significant reduction from the preoprerative values (*P* < 0.05). The preoperative mean spherical error was −3.20 ± 1.46 D (range −1.25−6.25) and changed to −2.73 ± 1.56 D (range −0.5−6.0). Ten eyes (66.67%) had reduction of spherical error of 0.50 D or more.

### Cylindrical error

There was no statistically significant change from the preoperative values (*P* > 0.05). The preoperative mean cylindrical error was 4.90 ± 0.74 D (range 3.75–6.0) and changed to 4.95 ± 0.76 D (range 4.0–06.0).

### Keratometry

There was a statistically significant decrease in the mean K readings from the preoperative values (*P* < 0.05). The preoperative mean K was 49.97 ± 2.81 D (range 47.20–51.75) and changed to 48.34 ± 2.64 D (range 45.75–50.40).

Figure 1

**Figure 1:**
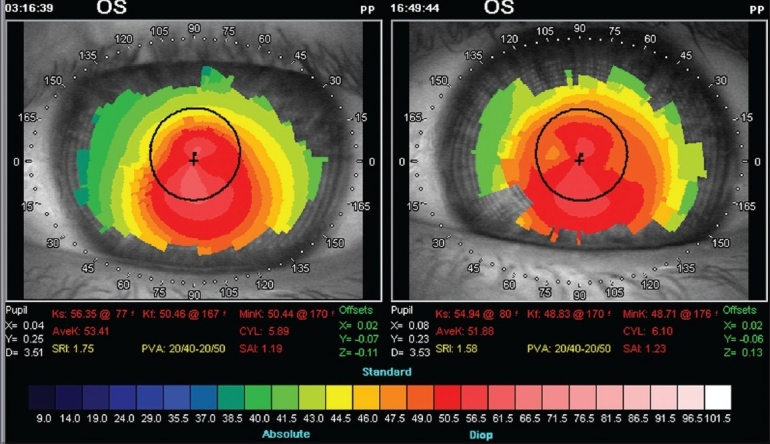
Topographic changes: Preoperative (left), postoperative (right). Notice the reduction of K readings and regularization of corneal shape with apparent shifting of cone apex towards corneal center

shows the topographic changes with reduction of K readings and regularization of corneal shape.

In the sixth month, there were no statistically significant changes in K readings, compared to third month records, denoting the short term stability of the topographic changes. Progression of corneal steepening from the preoperative value was not observed in any of the treated eyes.

### Pachymetry

There was no statistically significant change in corneal thickness from the preoperative values (*P* > 0.05). The preoperative mean pachymetry was 444 ± 18.42 u (range 420–480) and changed to 446.67 ± 18.39 u (range 420–480).

### Complications

All eyes developed faint diffuse stromal haze that cleared in 14 eyes (93.33%) within 1 month. In only 1 eye (6.67%) this resulted in a very faint superficial corneal scar that resulted in significant flattening of the K reading and 1 line improvement in both UCVA and BSCVA. Other sight threatening complications were not encountered in this series.

## DISCUSSION

Wollensak *et al*. introduced corneal collagen cross-linking for the treatment of progressive keratoconus in the year 2003. The technique was shown to arrest progression of keratoconus due to increase in biomechanical strength of the human cornea by approximately 300%.^9^,^10^,^14^

Cross-linking of corneal collagen is a new method to increase stability of the cornea by inducing additional cross-links between or within collagen fibers using UVA light and riboflavin as photomediators.^15^ Our results show statistically significant reduction in keratometric readings; revealing that riboflavin-UVA corneal cross-linking could partially reverse keratoconus. The observed reduction in K values was probably the result of the increased biomechanical stability of the cornea after cross-linking.^9^

There was a statistically significant reduction in mean K reading in this series - a decrease of 1.63 ± 0.17 D. This finding was also addressed by Caporossi * et al*., who recorded topographic mean reduction in dioptric power of 2.1 ± 0.13 D in the central 3.0 mm.^16^ Caporossi *et al*. noticed a trend towards a more regular corneal surface accompanied by an increase in the visual acuity after cross-linking in primary keratoconus;^16^ an effect also found in our patients. There was statistically significant improvement of UCVA. In 5 eyes there was 1 line improvement in UCVA while 4 eyes had 1 line improvement in BSCVA. The cause of this optical improvement is unknown.

It could be hypothesized that uneven thickness of the cornea means that thinner weak bulged areas of the cornea probably react more in the direction of restoration of its original shape than the thicker parts which are basically unchanged. This would explain why the cone seems to shift back towards the corneal center as noticed in Figure 1.

Although there was statistically significant reduction in keratometric readings, no significant reduction of the cylindrical error was noted. This finding may be related to the nearly equal reduction of both flat and steep corneal meridians which resulted in significant reduction of the spherical error without significant change in the cylindrical error.

Sight threatening complications were not encountered in this series denoting the safety of this procedure. All eyes developed faint diffuse stromal haze which cleared in 14 eyes within 5 weeks. In only 1 eye this resulted in a very faint superficial corneal scar causing significant flattening of the K reading and one line improvement in both UCVA and BSCVA.

In conclusion, riboflavin-UVA corneal cross-linking is a safe measure to increase stability of the cornea and may arrest or even reverse the progression of keratoconus at least in the short term as demonstrated by this study. This method is technically simpler, cheaper and less invasive than other therapies proposed for keratoconus. Cross-linking could be performed in many patients with non advanced keratoconus. Further studies with more patients and longer follow-up to verify the stability of the induced effect are recommended.
